# Evaluation design of Urban Health Centres Europe (UHCE): preventive integrated health and social care for community-dwelling older persons in five European cities

**DOI:** 10.1186/s12877-017-0606-1

**Published:** 2017-09-11

**Authors:** Carmen B. Franse, Antonius J.J. Voorham, Rob van Staveren, Elin Koppelaar, Rens Martijn, Elisa Valía-Cotanda, Tamara Alhambra-Borrás, Tasos Rentoumis, Lovorka Bilajac, Vanja Vasiljev Marchesi, Tomislav Rukavina, Arpana Verma, Greg Williams, Gary Clough, Jorge Garcés-Ferrer, Francesco Mattace Raso, Hein Raat

**Affiliations:** 1 Department of Public Health, Erasmus University Medical Center, Wytemaweg 80, 3015 CN Rotterdam, The Netherlands; 2 Rotterdam University of Applied Sciences, Research Centre Innovation in Care, Rotterdam, The Netherlands; 3 Zorg Op Noord, Capelle aan den IJssel, The Netherlands; 4 Polibienestar Research Institute, University of Valencia, Valencia, Spain; 5 Vidavo Health Telematics, Thessalonikis, Greece; 6 Department of Social Medicine and Epidemiology, Faculty of Medicine University of Rijeka, Rijeka, Croatia; 7 Teaching institute of Public Health Primorsko-goranska County, Branch Office Opatija, Rijeka, Croatia; 8 Department of Public Health, Faculty of Health Studies, University of Rijeka, Rijeka, Croatia; 9 Manchester Urban Collaboration on Health, Centre for Epidemiology, Division of Population Health, Health Services Research and Primary Care, Manchester Academic Health Science Centre, The University of Manchester, Manchester, UK; 10 Erasmus University Medical Center, Section of geriatric medicine, department of Internal Medicine, Rotterdam, The Netherlands

**Keywords:** Integrated health and social care, Prevention, Frailty, Older citizens, Primary care, Specific pre-post controlled clinical trial

## Abstract

**Background:**

Older persons often have interacting physical and social problems and complex care needs. An integrated care approach in the local context with collaborations between community-, social-, and health-focused organisations can contribute to the promotion of independent living and quality of life. In the Urban Health Centres Europe (UHCE) project, five European cities (Greater Manchester, United Kingdom; Pallini (in Greater Athens Area), Greece; Rijeka, Croatia; Rotterdam, the Netherlands; and Valencia, Spain) develop and implement a care template that integrates health and social care and includes a preventive approach. The UHCE project includes an effect and process evaluation.

**Methods:**

In a one-year pre-post controlled trial, in each city 250 participants aged 75+ years are recruited to receive the UHCE approach and are compared with 250 participants who receive ‘care as usual’. Benefits of UHCE approach in terms of healthy life styles, fall risk, appropriate medication use, loneliness level and frailty, and in terms of level of independence and health-related quality of life and health care use are assessed. A multilevel modeling approach is used for the analyses. The process evaluation is used to provide insight into the reach of the target population, the extent to which elements of the UHCE approach are executed as planned and the satisfaction of the participants.

**Discussion:**

The UHCE project will provide new insight into the feasibility and effectiveness of an integrated care approach for older persons in different European settings.

**Trial registration:**

ISRCTN registry number is ISRCTN52788952. Date of registration is 13/03/2017.

## Background

By 2040, Europeans over 65 years old will account for 27% of the EU-28’s population, compared with 18% in 2013, according to Eurostat predictions [1]. This will be associated with a steep increase in demand for care. Health professionals, including physicians, will have an increasing workload and have limited time for prevention. Adding to this, older persons often have multiple, interacting social and health problems [2–4]. However, care is currently often characterised by a monodisciplinary approach, where health and social care are isolated from each other [5, 6].

A preventive integrated care approach in the local context where informal and formal infrastructures can be connected, and where community-, social-, and health-focused organisations are collaborating can contribute to the promotion of independent living and quality of life [7, 8]. A care coordinator, typically a nurse practitioner or physician assistant, may play a key role in assessing physical and social problems among older people and coordinate follow-up care [9–12]. This promotes collaboration and communication between community-, social-, and health-focused professionals. However, more insight is still needed in ways to combine health and social care and the content of effective care pathways [7]. Previous research efforts on integrated care have had mixed results and best practices are needed [13, 14]. Although there is evidence that physical exercise programs contribute to better health and prevent falls in older populations [15–17], less is known about the effects of social care programs when integrated in health care [18–20]. Integration between social and health care can be organized in different ways according to availability and organizational structures in the local context, therefore it is valuable to evaluate the effectiveness of integrated care approaches in different (international) settings.

### The UHCE project

In the Urban Health Centres Europe (UHCE) project, a consortium of twelve European partners was set up to respond to the call of the European Commission Executive Agency for Health and Consumers to improve and evaluate community action in the field of health, in particular the improvement of management of multi-morbidity of older persons using integrated care pathways that focus on adherence to treatment and prevention of falls and frailty. (www.uhce.eu). UHCE aims to address three pertinent issues among older persons; (a) appropriate medication prescription and adherence, (b) falls prevention, (c) prevention of functional decline and frailty. In this project, five European cities (Greater Manchester, United Kingdom; Pallini, Greece; Rijeka, Croatia; Rotterdam, the Netherlands; and Valencia, Spain) will develop and implement a care template that integrates health and social care and includes a population oriented, preventive approach. In UHCE, a general care template is adapted to the local context of the five cities. The main objective of the evaluation study is to evaluate the UHCE approach in a pre-post controlled design in terms of benefits for older persons (75 years and older) involved and process performance. The following research questions will be answered:

1. What are the benefits of the UHCE approach for older persons in terms of healthy life styles, fall risk, appropriate medication use, loneliness and frailty, as well as the benefits in terms of level of independence and health-related quality of life?
2. What are the benefits of the UHCE approach in terms of reducing the use of ambulatory and residential health- and social care among older persons?
3. What is the reach of the target population by the UHCE approach, to what extent are the elements of the UHCE template executed as planned and are the main stakeholders satisfied?

The UHCE approach is applied in an intervention group, which is compared with a control group. We hypothesize that intervention group participants, compared to those in the control group have more favourable life styles (physical activity, smoking, alcohol use) less fall risk and higher appropriate medication use, less loneliness, less frailty, higher level of independence and more favourable health-related quality of life. We furthermore hypothesize that participants in the intervention group, compared to the control group use less ambulatory, residential and social care. We aim towards a reach of participants in the intervention group of 70% or higher, and an appreciation of 7 or higher on a 1–10 scale.

## Methods/design

### The intervention: The UHCE approach

A general template of the UHCE approach was developed by systematically reviewing the literature to identify evidence based interventions and validated assessment instruments for frailty, fall risk and polypharmacy (see www.uhce.eu). Furthermore, focus groups and interviews with main stakeholders (older persons, health and social care professionals, caregivers and policy makers) were held to identify their demands and preferences regarding the UHCE template, which led to the decision to address loneliness as a separate health problem, in addition to frailty, fall risk and polypharmacy.

**Fig. 1:**
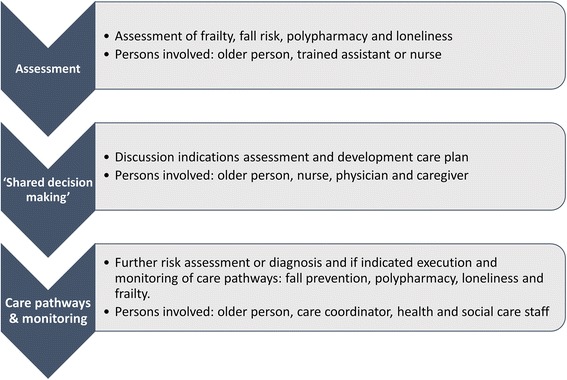
The UHCE approach

UHCE starts with a frailty assessment and an assessment of fall risks, polypharmacy and loneliness in order to identify priorities for prevention and care of the older persons participating (Fig. 1). We use validated instruments that are practical and commonly used in a primary care setting. *Fall risk is measured following a validated protocol developed by the Dutch safety research institute* [21]*. Polypharmacy is measured following the common definition of using of five or more different medicines* [22]*, in addition we measure whether persons have difficulty to take the medicines as prescribed* [23]*. Loneliness is measured with the social subscale of the Tilburg Frailty Indicator* [24]*. Frailty is measured with the Tilburg Frailty indicator, which was made and validated for use in primary care and has been extensively researched* [24, 25]*.*

The results of the assessments are discussed with the older person, a person in charge of care coordination (nurse practitioner or other) and a physician (Fig. 1). As a result of this shared decision-making process, a decision on a care plan is made and each participant is referred to evidence based “care pathways” (interventions) that are described in the UHCE template and adapted to the context of each of the five participating cities. The main UHCE care pathways are: multifactorial fall prevention actions (which include physical exercise groups, home hazard identification or other actions based on the judgement of a physician), actions addressing polypharmacy (which include appropriate prescribing and adherence action or other actions based on the judgement of a physician), actions addressing loneliness (which include support groups, social activities, or other actions based on the judgement of a physician) and frailty action (which include group based exercise programs or other actions based on the judgement of a physician).

The care coordinator (a nurse, social worker or trained physician assistant) coordinates and monitors the progress of each individual care plan under the supervision of the physician (Fig. 1). Follow-up visits are scheduled if needed. The care coordinator monitors the compliance to the care plan. The general UHCE template is adjusted in accordance with national standards and the local setting of the five participating cities.

### Design, setting and procedures

The evaluation of UHCE has a specific pre-post controlled design [26]. Intervention and control sites (general practitioner; GP practices or primary health centres; PHC) are chosen based on their location in distinct neighbourhoods in the participating cities. Older persons in the catchment area of an intervention site receive an invitation by their physician to join the study in the area where the UHCE approach is applied. Older persons in the catchment area of a control site receive an invitation by their physician to join the study in the area where ‘usual care’ is applied. The study is performed in accordance with the capacity, organizational and contextual factors of each of the five participating cities, as described below.

*Greater Manchester* is a metropolitan county in North West England, with a population of 2.7 million persons [27]. Participants are recruited through GPs of individual GP practices, the intervention practices are located in Tameside and Glossop districts and the control practices are located in South Manchester. Assessments are taken at the participant’s home by a trained assistant. The results are assessed by researchers and clinicians before being provided to the participant’s GP. The participant’s GP is responsible for decisions on care in collaboration with health and social care staff at the GP practice.

*Pallini* is a suburban town and a municipality situated in the eastern part of the greater Athens area, with a population of 54,415 persons [28]. Participants are recruited through the Municipal Health and Social Services. In Pallini, as only city, participants are randomized (by using a random numbers table) into the intervention group (UHCE approach) and the control group (‘usual care’). Assessments are taken at three community centres and the Municipal Health Centre by trained health staff. A health professional or a social worker is the care coordinator, and a physician is responsible for decisions on care in collaboration with the nurse.

*Rijeka* is a port city located at the most western part of the Republic of Croatia and has a population of 128,384 persons [29]. Participants are recruited through individual GP practices, intervention practices are located in the Western part of Rijeka and control practices are located in the Eastern part of Rijeka. Assessments are taken at the participant’s home by community nurses, who act as care coordinators. The participant’s GP, supported by the nurse is responsible for decisions on care.

*Rotterdam* is a port city in the Netherlands province of South Holland, with a population of 638,714 persons [30]. Participants are recruited through their GP based in PHC, the intervention PHC is located in Ommoord neighbourhood and the control PHCs are located in the Oosterflank and Zevenkamp neighbourhoods. Assessments are taken at the participant’s home by a trained assistant. Results are then provided to a geriatric nurse, who is the care coordinator, in collaboration with the GP. The participant’s GP, supported by the nurse is responsible for decisions on care.

*Valencia* is a port city located on the Southeastern coast of Spain, it has a population of 800,666 persons [31]. Participants are recruited through the GPs of the intervention PHC in the Nou Moles neighbourhood and the control PHC in the El Botanic neighbourhood. Assessments are taken at the participant’s home by a trained assistant who supports case management by the GP. The participant’s GP is responsible for decisions on care supported by the nurse and social workers of the health centres.

### Study population and eligibility to participate in the study

We aim to include 250 participants in both the intervention group and 250 participants in the control group in each city. In total, 1250 participants are included in the intervention group and 1250 participants in the control group. In each city, the target population consists of persons living independently, aged 75 years or more, who are, according to their GP, expected to be able to participate in the study for at least 6 months. Persons are not eligible to participate if they are not able to comprehend the information provided in the local language or if they are not able to cognitively evaluate the risks and benefits of participation and are not expected to be able to make an informed decision regarding participation in the study, according to their GP or physician. If possible, the participant is invited to designate an informal caregiver to support him or her, such as the partner, a child, sibling, friend or neighbour.

### Data-collection and measures

Data collection is done with the use of a questionnaire; which includes the UHCE assessment (described above), outcome and other measures. Two non-invasive measurements (hand-grip strength and mid-upper arm circumference) are additionally performed and written down in the questionnaire. These data are collected at both baseline (T0) and after 12 months (T1). The instruments used for the outcome measures are described in the measurements section. The instruments and items for which no validated translations are available are translated forward and backward by translators. Forward- and back-translations are discussed by the study team and translation is adapted when needed.

#### Outcome measures

Both general outcome health measures and specific outcome health measures applicable to each care pathway are applied: healthy life styles, fall risk, appropriate medication use, loneliness, frailty, level of independence and health-related quality of life. Healthy life style is measured with one item on physical activity, two items on smoking, and three items of the AUDIT-C [32] on high-risk alcohol use. Fall risk is measured by two items on (the number of) falls in the previous year, a single item asking whether or not the person is afraid of falling, and fear of falling while performing several daily activities as measured by the 7-item Falls Efficacy Scale International (FES-I) short version [33]. Appropriate medication use is measured with 10 items of the Medication risk questionnaire (MRQ-10) [23], a tool developed for use by older persons to identify who is at increased risk of potentially experiencing a medication-related problem. Loneliness is measured with the short 6-item version of the Jong Gierveld loneliness scale [34], which measures the degree of what one wants and what one has in terms of interpersonal affection and intimacy. Frailty is measured with the 15-item Tilburg Frailty indicator [24, 25], that includes questions on physical, psychological and social components of frailty. Physical frailty is additionally measured with the SHARE-Frailty instrument which is an instrument that was developed and validated in a European population [35, 36], SHARE-frailty includes hand-grip strength measurement, and physical frailty is also measured with a measurement of the mid-upper arm circumference [37], a measure for malnutrition. Level of independence is measured with the Groningen activity restriction scale [38], that includes 18 items on independence of activities of daily living (ADL) and instrumental activities of daily living (IADL) and additionally with the one-item Global Activity Limitation Index (GALI) [39, 40]. Health-related quality of life is measured with the 12-item short-form (SF-12) [41, 42] and the full 5-item mental health scale of the SF-36 [43].

Additionally to health measures, use of care is measured with four questions regarding the use of doctor appointments, household work, help caring (such as washing or dressing) and hospital admissions.

#### Other measures

Various socio-demographic characteristics are measured: age, gender, country of birth, educational level, income, marital status, employment situation, household composition and religion. Additionally, several questions on the participant’s general health are asked: self-reported height and weight, use of walking or other aids, whether they ever have been diagnosed with any of fifteen listed health conditions. Any additional remarks can be left in an open box at the end of the questionnaire.

### Process evaluation

A process evaluation is used to monitor program implementation and help to understand the relationship between the delivery of specific UHCE approach elements and program outcomes. We based our design of the process evaluation on the theoretical framework for public health interventions as developed by Steckler and Linnan [44, 45]. The following elements are included and outlined below: reach, dose delivered and received, fidelity, satisfaction, and context.

#### Reach

This process element aims at measuring the proportion of the intended target population that is reached by the care approach. In UHCE we calculate which proportion of the participants that we contacted participate in the UHCE approach. If possible reasons for refusal are reported.

#### Dose delivered and received

Dose delivered measures whether the anticipated care is offered to the participant and dose received measures the extent in which participants actively engage in the care that is offered. For this purpose, data on the delivery of the UHCE approach and reasons for non-participation in care are recorded by the care coordinator. A 1-page logbook is kept for each participant that includes the different stages of the UHCE approach: 1. assessment, 2 shared decision making, and 3. care pathways and monitoring. At least after 6 months, the care coordinator records (if needed telephone contact with the participant or responsible health care provider is sought) whether key elements of the UHCE approach are delivered. In case of loss to follow- up at the T1 measurement, reasons are recorded by the research staff, given they are provided by the participant.

#### Fidelity and satisfaction

We aim to measure the extent to which the UHCE approach is implemented as planned (fidelity) and the satisfaction of main stakeholders with the UHCE approach. In the T1 questionnaire, three items measure the general satisfaction with professional, social and self-care in the past 12 months, 4 items measure the satisfaction with specific UHCE elements (assessment, shared-decision making and care-pathways) and a final question rates the whole UHCE approach on a scale from 1 to 10. To gain more in-depth knowledge on how the UHCE approach is carried out and which barriers are encountered, focus groups and semi-structured interviews with older persons, caregivers, and social and health professionals involved in UHCE are held 12 months after inclusion of the last group of participants. In each city we organize 1 focus group with 6–8 older persons and caregivers (e.g. family, friends) and 1 group with 6–8 social and health care professionals. All professionals involved with UHCE are invited to participate in the focus groups. The focus group discussions are recorded and translated in English.

#### Context

As the UHCE approach is implemented in five diverse settings its’ success depends on the context in which it is implemented. With the use of structured forms we make an inventory of relevant contextual factors of each city in which the UHCE approach is embedded; type and experience of health staff, setting, resources and interventions available or newly developed.

### Power considerations

In each of the five cities, 250 participants are included in the intervention group and 250 participants in the control group. Assuming a 20% loss to follow-up between T0 and T1 due to mortality, rehousing or impossibility to participate, we expect to receive complete data of 2000 participants at follow up, equally divided over the intervention group and the control group. We assume equal standard deviations in the intervention group and the control group, alpha of 0.05 and power of 0.80. Given 5 participating cities with each an intervention and control group, we applied a correction factor to account for the cluster design, assuming an average cluster size of 200 older citizens (2000/10) and an intra-class correlation coefficient of 0.02. For this expected sample size and assumptions, with regard to the continuous outcome measures, a difference of 0.25 SD (standard deviation) between the intervention and the control group can be detected at follow-up. For example, regarding the health-related quality of life as measured by the Physical and Mental Component Summary Scale scores of the SF-12, a difference of 2.9 points can be detected for the Physical Component Summary Scale (SD = 11.4) and 3.0 points for the Mental Component Summary Scale (SD = 11.9) [46].

### Data management and analysis

Data from all cities is combined and data-management and analysis is done at Erasmus MC. Paper questionnaires are scanned and automatically transferred into electronic files. Paper participant logbooks are entered into an electronic data-entry form by research staff. Electronic data is checked for missing or incorrect data. If an error is present in the electronic data, scans of the paper questionnaires and logbooks are consulted. If needed responsible staff are contacted for clarification.

Descriptive statistics are used to summarize characteristics of participants in each city and in the total study population. Participant socio-demographic characteristics (age, gender, income, educational level) are compared at baseline between the intervention and control group of each city and in the total study population. A multilevel modelling approach is used to examine differences in the outcome measures between the intervention and control group, taking into account the clustering effects at the city-level. Multilevel linear regression analyses are conducted for the continuous outcome variables with group (intervention or control) as independent variable and baseline values and potential confounders as covariates. Multilevel logistic regression is performed for dichotomous outcome variables. Subgroup analyses are conducted by means of formal interaction tests for intervention and those variables which are likely to influence the effect of the intervention itself: gender, age and educational level. In addition, subgroup analyses are done for subgroups of individuals with an indication for specific care pathways (frailty, fall risk, polypharmacy and loneliness), comparing participants with this indication in control and intervention groups. In addition, the above mentioned analyses are repeated for each city separately.

All qualitative data (interviews and focus groups) are recorded, transcribed and translated to English. Thematic analysis of data is done using a pre-defined coding framework which is developed through discussion and consensus among the research team [47].

### Dissemination

We have set up an Advisory Board with experts from six EU countries. The role of the Advisory Board is to provide a critical perspective throughout the project. The scientific project results are disseminated by the project team through publications in scientific journals and conferences. To further disseminate the knowledge to all stakeholders we use the project website (www.uhce.eu). The European Local Inclusion and Social Action Network (ELISAN) is one of the partners of the UHCE project and aids the dissemination of project results to all stakeholders via social media.

## Discussion

This study aims to evaluate the potential benefits of the UHCE approach on healthy lifestyle, fall risks, appropriate medication use, loneliness, frailty, level of independence and quality of life in older European persons. This is done using a pre-post controlled design in five European cities: (Greater Manchester, United Kingdom; Pallini (in Greater Athens Area), Greece; Rijeka, Croatia; Rotterdam, the Netherlands; and Valencia, Spain). This study has several strengths. To our knowledge this is one of the first European studies that aims to implement an integrated care approach in different European settings. Carrying out an integrated care approach in different settings will provide information on the generalizability of the care approach in various European settings. This could also help facilitate future implementation into routine primary care practice. The development of the UHCE approach was based on the experiences and preferences of a diverse group of stakeholders (older persons, their caregivers, medical and social care providers), which supported the co-creation of the intervention for the end-user and could generate a wider acceptance of the intervention.

The proposed study has some limitations and we expect to encounter some challenges. Participation of older persons may be a problem; that may affect the cities differently. To increase participation, requests to participate are sent through their personal GP, where possible. Because our target group consists of older persons, we also expect persons to move to another place (e.g. nursing home), pass away during the follow-up period, or not be fit enough to participate. We apply a non-randomized design, which makes results subject to confounding variables. Randomization was not desirable for cities that worked with GP practices as it is not feasible for GPs to give ‘usual care’ and care according to UHCE at the same time. In our questionnaire we tried to capture the most important confounding variables; however it remains possible that we missed other relevant variables.

As the growth of the European older population will pose a challenge for the European Union, new ways of providing care are necessary. Integrating social and health care and providing a preventive care approach may provide better outcomes. The UHCE project will further elucidate whether such an approach could be effective and feasible for an older population in different settings and identify potential effective elements of integrated preventive care.

## Abbreviations

ADL: Activities of daily living
AUDIT-C: The alcohol use disorders identification test
ELISAN: European local inclusion and social action network
EU: European Union
FES-I: Falls Efficacy Scale International
GALI: Global activity limitation index
GP: General practitioner
IADL: Instrumental activities of daily living
MRQ-10: Medication risk questionnaire
PHC: Primary health centre
SF-12: Short-form
SHARE: Survey of Health, Ageing and Retirement in Europe
UHCE: Urban Health Centres Europe

## References

[1] Eurostat 2016, Population structure and ageing. http://epp.eurostat.ec.europa.eu/statistics_explained/index.php/Population_structure_and_ageing.

[2] Andrew MK, Mitnitski A, Kirkland SA, Rockwood K (2012). The impact of social vulnerability on the survival of the fittest older adults. Age Ageing.

[3] Fratiglioni L, Wang HX, Ericsson K, Maytan M, Winblad B (2000). Influence of social network on occurrence of dementia: a community-based longitudinal study. Lancet.

[4] Mendes de Leon CF, Glass TA, Berkman LF (2003). Social engagement and disability in a community population of older adults: the New Haven EPESE. Am J Epidemiol.

[5] Hunt L (2014). Test and learn: working towards integrated services. Nurs Older People.

[6] Glasby J (2017). The holy grail of health and social care integration. BMJ.

[7] Eklund K, Wilhelmson K (2009). Outcomes of coordinated and integrated interventions targeting frail elderly people: a systematic review of randomised controlled trials. Health Soc Care Community.

[8] van Leeuwen KM, Bosmans JE, Jansen AP, Hoogendijk EO, Muntinga ME, van Hout HP, Nijpels G, van der Horst HE, van Tulder MW (2015). Cost-effectiveness of a chronic care model for frail older adults in primary care: economic evaluation alongside a stepped-wedge cluster-randomized trial. J Am Geriatr Soc.

[9] Frich LM (2003). Nursing interventions for patients with chronic conditions. J Adv Nurs.

[10] Markle-Reid M, Browne G, Weir R, Gafni A, Roberts J, Henderson SR (2006). The effectiveness and efficiency of home-based nursing health promotion for older people: a review of the literature. Med Care Res Rev.

[11] Markle-Reid M, Browne G, Gafni A (2013). Nurse-led health promotion interventions improve quality of life in frail older home care clients: lessons learned from three randomized trials in Ontario. Canada J Eval Clin Pract.

[12] Liebel DV, Friedman B, Watson NM, Powers BA (2009). Review of nurse home visiting interventions for community-dwelling older persons with existing disability. Med Care Res Rev.

[13] Metzelthin SF, van Rossum E, de Witte LP, Ambergen AW, Hobma SO, Sipers W, Kempen GI (2013). Effectiveness of interdisciplinary primary care approach to reduce disability in community dwelling frail older people: cluster randomised controlled trial. BMJ.

[14] Janse B, Huijsman R, de Kuyper RD, Fabbricotti IN (2014). The effects of an integrated care intervention for the frail elderly on informal caregivers: a quasi-experimental study. BMC Geriatr.

[15] El-Khoury F, Cassou B, Charles MA, Dargent-Molina P (2013). The effect of fall prevention exercise programmes on fall induced injuries in community dwelling older adults: systematic review and meta-analysis of randomised controlled trials. BMJ.

[16] Gillespie LD, Robertson MC, Gillespie WJ, Lamb SE, Gates S, Cumming RG, Rowe BH (2009). Interventions for preventing falls in older people living in the community. Cochrane Database Syst Rev.

[17] Howe TE, Rochester L, Neil F, Skelton DA, Ballinger C (2011). Exercise for improving balance in older people. Cochrane Database Syst Rev.

[18] Cattan M, White M, Bond J, Learmouth A (2005). Preventing social isolation and loneliness among older people: a systematic review of health promotion interventions. Ageing Soc.

[19] Hagan R, Manktelow R, Taylor BJ, Mallett J (2014). Reducing loneliness amongst older people: a systematic search and narrative review. Aging Ment Health.

[20] Dickens AP, Richards SH, Greaves CJ, Campbell JL (2011). Interventions targeting social isolation in older people: a systematic review. BMC Public Health.

[21] VeiligheidNL 2014, Fall risk test. https://www.veiligheid.nl/valpreventie/interventies/screening/valanalyse.

[22] Maher RL, Hanlon J, Hajjar ER (2014). Clinical consequences of polypharmacy in elderly. Expert Opin Drug Saf.

[23] Barenholtz Levy H (2003). Self-administered medication-risk questionnaire in an elderly population. Ann Pharmacother.

[24] Gobbens RJ, van Assen MA, Luijkx KG, Wijnen-Sponselee MT, Schols JM (2010). The Tilburg frailty indicator: psychometric properties. J Am Med Dir Assoc.

[25] Gobbens RJ, Luijkx KG, Wijnen-Sponselee MT, Schols JM (2010). Towards an integral conceptual model of frailty. J Nutr Health Aging.

[26] Miller JN, Colditz GA, Mosteller F (1989). How study design affects outcomes in comparisons of therapy. II: Surgical. Stat Med.

[27] Office for National Statistics 2017, Population Estimates for UK, England and Wales, Scotland and Northern Ireland, mid-2014 population estimates. https://www.ons.gov.uk/peoplepopulationandcommunity/populationandmigration/populationestimates/datasets/populationestimatesforukenglandandwalesscotlandandnorthernireland.

[28] Hellenic Statistical Authority 2011, Population-Housing Census. http://www.statistics.gr/en/statistics/-/publication/SAM03/-.

[29] Croatian Bureau of Statistics 2011, Census of Population, Households and Dwellings. http://www.dzs.hr/default_e.htm.

[30] Statline. 2017. http://statline.cbs.nl/Statweb/dome/?TH=3600&LA=nl.

[31] Statistics Office Valencia 2016, Summary of the city of Valencia. http://www.valencia.es/ayuntamiento/webs/estadistica/Recull/RECULL2016_Ingles.pdf.

[32] Bush K, Kivlahan DR, McDonell MB, Fihn SD, Bradley KA (1998). The AUDIT alcohol consumption questions (AUDIT-C): an effective brief screening test for problem drinking. Ambulatory care quality improvement project (ACQUIP). Alcohol use disorders identification test. Arch Intern Med.

[33] Yardley L, Beyer N, Hauer K, Kempen G, Piot-Ziegler C, Todd C (2005). Development and initial validation of the falls efficacy scale-international (FES-I). Age Ageing.

[34] De Jong GJ, Van Tilburg T (2010). The de Jong Gierveld short scales for emotional and social loneliness: tested on data from 7 countries in the UN generations and gender surveys. Eur J Ageing.

[35] Romero-Ortuno R, Walsh CD, Lawlor BA, Kenny RA (2010). A frailty instrument for primary care: findings from the survey of health, ageing and retirement in Europe (SHARE). BMC Geriatr.

[36] Romero-Ortuno R (2013). The frailty instrument for primary care of the survey of health, ageing and retirement in Europe predicts mortality similarly to a frailty index based on comprehensive geriatric assessment. Geriatr Gerontol Int.

[37] Wijnhoven HA, Schilp J, van Bokhorst-de van der Schueren MA, de Vet HC, Kruizenga HM, Deeg DJ, Ferrucci L, Visser M (2012). Development and validation of criteria for determining undernutrition in community-dwelling older men and women: the short nutritional assessment questionnaire 65+. Clin Nutr.

[38] Suurmeijer TP, Doeglas DM, Moum T, Briancon S, Krol B, Sanderman R, Guillemin F, Bjelle A, van den Heuvel WJ (1994). The Groningen activity restriction scale for measuring disability: its utility in international comparisons. Am J Public Health.

[39] van Oyen H, Van der Heyden J, Perenboom R, Jagger C (2006). Monitoring population disability: evaluation of a new global activity limitation indicator (GALI). Soz Praventivmed.

[40] Berger N, Van Oyen H, Cambois E, Fouweather T, Jagger C, Nusselder W, Robine JM (2015). Assessing the validity of the global activity limitation indicator in fourteen European countries. BMC Med Res Methodol.

[41] Haywood KL, Garratt AM, Fitzpatrick R (2005). Quality of life in older people: a structured review of generic self-assessed health instruments. Qual Life Res.

[42] Ware J, Kosinski M, Keller SD (1996). A 12-item short-form health survey: construction of scales and preliminary tests of reliability and validity. Med Care.

[43] Ware JE, Sherbourne CD (1992). The MOS 36-item short-form health survey (SF-36). I. Conceptual framework and item selection. Med Care.

[44] Steckler ABLL: Process evaluation for public health interventions and research. San Francisco, Calif.: Jossey-Bass; 2002.

[45] Saunders RP, Evans MH, Joshi P (2005). Developing a process-evaluation plan for assessing health promotion program implementation: a how-to guide. Health Promot Pract.

[46] Aaronson NK, Muller M, Cohen PD, Essink-Bot ML, Fekkes M, Sanderman R, Sprangers MA, te Velde A, Verrips E (1998). Translation, validation, and norming of the Dutch language version of the SF-36 health survey in community and chronic disease populations. J Clin Epidemiol.

[47] Boyatzis RE: Transforming qualitative information: thematic analysis and code development. Thousand Oaks [u.a.]: Sage; 2009.

